# Dietary *Bacillus velezensis* Improves Piglet Intestinal Health and Antioxidant Capacity via Regulating the Gut Microbiota

**DOI:** 10.3390/ijms26125875

**Published:** 2025-06-19

**Authors:** Linbao Ji, Jiakun Shen, Chunchen Liu, Junshu Yan, Xi Ma

**Affiliations:** 1 State Key Laboratory of Animal Nutrition and Feeding, College of Animal Science and Technology, China Agricultural University, Beijing 100193, China; jilinbao0126@cau.edu.cn (L.J.); shenjiakun2008@126.com (J.S.); liuchunchenlcc@163.com (C.L.); 2 Institute of Animal Science, Jiangsu Academy of Agricultural Sciences, Nanjing 210014, China

**Keywords:** piglets, dietary supplementation, probiotics, *Bacillus velezensis*, diarrhea, gut microbiota

## Abstract

Piglet diarrhea caused by weaning stress will increase the mortality rate and seriously affect swine industry production efficiency. Probiotic supplementation has been reported to effectively alleviate weaning diarrhea by inhibiting the colonization of pathogenic microorganisms; however, the underlying mechanisms remain unclear. In this study, we isolated a strain of *Bacillus velezensis* and conducted a series of *in vivo* and *in vitro* experiments to explore its effects on weaned piglets. The piglets were fed for a 28-day period, and the results showed that dietary supplementation of *B. velezensis* 411 significantly alleviated weaning diarrhea (*p* = 0.019) and improved the average daily gain (ADG) of piglets throughout the experimental period (*p* = 0.004). The intestinal antioxidant capacity of piglets was also significantly enhanced. Whole-genome sequencing revealed that *B. velezensis* 411 contains a protein-encoding circular chromosome, which is involved in biological processes such as sporulation and antibiotic secretion. Supplementation with *B. velezensis* 411 significantly increased the abundance of *Akkermansia* in intestine samples and significantly decreased the abundance of pathogenic bacteria, including *Escherichia coli* and *Staphylococcus aureus*, in piglets (*p* < 0.05). The transcriptomic results suggest that *B. velezensis* 411 supplementation may alter the composition of intestinal microorganisms through regulating the expression of *MPEG1*. Collectively, dietary *B. velezensis* can relieve diarrhea in piglets and improve their production performance by influencing the antioxidant capacity of the intestines and the balance of the intestinal flora. This study provides valuable insights into the potential application of *Bacillus velezensis* in mitigating weaning-associated issues in piglets.

## 1. Introduction

An early weaning strategy has been widely adopted in swine production, as it reduces the slaughter cycle and enhances the reproductive performance of sows [1]. For piglets, premature weaning leads to severe diarrhea, which can significantly impact their survival rates. For many decades, antibiotics used to play an important role in the prevention of diarrhea in early-weaned piglets for many decades [2]. The extensive use of antibiotics has led to a range of food safety issues. Consequently, several countries have implemented bans on the use of antibiotics in animal breeding; for instance, Sweden prohibited their use in animal husbandry in 1986, followed by the European Union in 2006 [3]. The Chinese government is now prohibiting the supplementation of antibiotics in feed. Therefore, it is urgent and essential to find alternatives to antibiotics in feed, preferably with the dual goal of effectively reducing piglet diarrhea while simultaneously promoting the growth of piglets [4].

Studies have reported the potential of *B. velezensis* and other probiotics for producing gastrointestinal beneficial effects and enhancing host health through modulation of the intestinal environment [5,6,7]. *Roseburia* spp., *Akkermansia* spp., *Propionibacterium* spp., and *Faecalibacterium* spp. show promise for the future [7] of probiotics, while *Lactobacillus*, *Bifidobacterium*, and *Saccharomyces* strains are the traditional dominant strains. Research studies have discovered that more and more probiotics are both safe and effective. It has been reported that Bacillus, including *Bacillus licheniformis*, *Bacillus subtilis*, and *Bacillus amylolyticus*, can produce various antimicrobial metabolites (such as lipopeptides), enzymes (such as proteases, amylases, and lipases), and biopeptides. These metabolites are effective in promoting the digestion and absorption of nutrients in the intestinal tract of animals, regulating microflora, promoting intestinal health, and improving the immunity of young livestock and poultry [8]. *Bacillus* species are extensively utilized in animal husbandry as probiotics, growth promoters, and a competition eliminator for animals. In aquaculture, *Bacillus* has been used to improve the growth and disease resistance of shrimp. Although *B. licheniformis*, *B. subtilis*, and *B. amyloliquefaciens* are the dominant species in the genus *Bacillus*, numerous studies have been focused on *B. velezensis* over the last decade [9]. *B. velezensis* is a Gram-positive aerobic, which can form endospores to promote plant growth and is widely used in agriculture [10]. Sub-strains of *B. velezensis* have been reported to inhibit the growth of microbial pathogens like bacteria, fungi, and nematodes [11]. The genome-wide sequencing project of *B. velezensis* has identified many biosynthetic gene clusters that encode antimicrobial compounds and the enzymes involved in their synthesis [12].

While studies have reported the potential of *B. velezensis* to improve gut health and immunity in mice [13], the mechanisms of action of *B. velezensis* on monogastric animals such as pigs and chickens are unclear. In this study, we discovered and isolated a strain of *Bacillus velezensis* and conducted a series of in vivo and in vitro experiments to explore its effects on weaned piglets. Piglets were fed for a 28-day period, and the results showed that dietary supplementation of *B. velezensis* 411 significantly alleviated weaning diarrhea and improved the average daily gain (ADG) of piglets throughout the experimental period. The objectives of this study were (1) to provide a complete genome sequence of *B. velezensis* 411; (2) to present data supporting the use of *B. velezensis* 411 as a feed additive; and (3) to investigate the effects of *B. velezensis* 411 on intestinal health in various mammalian species. The findings indicate that dietary supplementation of *B. velezensis* can relieve weaning diarrhea and inhibit pathogenic microorganisms, which may be mediated by improved intestinal antioxidant capacity and *MPEG1* expression. This study serves as reference for the application of *Bacillus velezensis* in weaned piglets.

## 2. Results

### 2.1. Colony Morphology and Prebiotic Potential of B. velezensis 411

**Figure 1:**
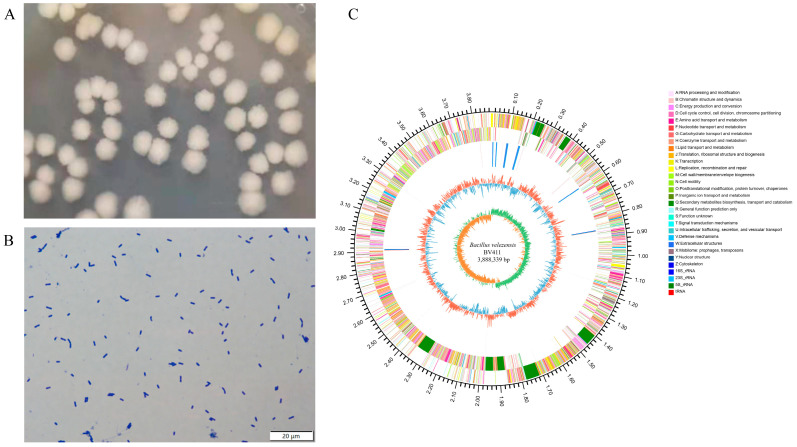
Isolation and identification of *B. velezensis* 411. (**A**) Colony morphology of *B. velezensis* 411. (**B**) Gram staining of *B. velezensis* 411; bar = 20 μm. (**C**) Whole genome of *B. velezensis* 411.

The morphology of the colonies was characterized by a white, rounded appearance with dry, wrinkled, opaque, and irregular edges on the surface (Figure 1A). The strain was examined using a 100 × oil microscope after being stained with crystal violet. The bacteria exhibited a rod-like form and ranged in size from 1 to 4 μm (Figure 1B). Genome sequencing results show the bacterial genome consists of a circular chromosome allele with a total length of 3,888,339 bp (Figure 1C). A total of 3662 genes were predicted, which was the number of the ORF (open reading frame). The total length of the ORF was 3,445,578 bp, the average length was 940.90 bp, and the GC content was 47.42% (Table A1). The strain is named *B. velezensis* 411.

### 2.2. Characteristics of B. velezensis 411 Growth

**Figure 2:**
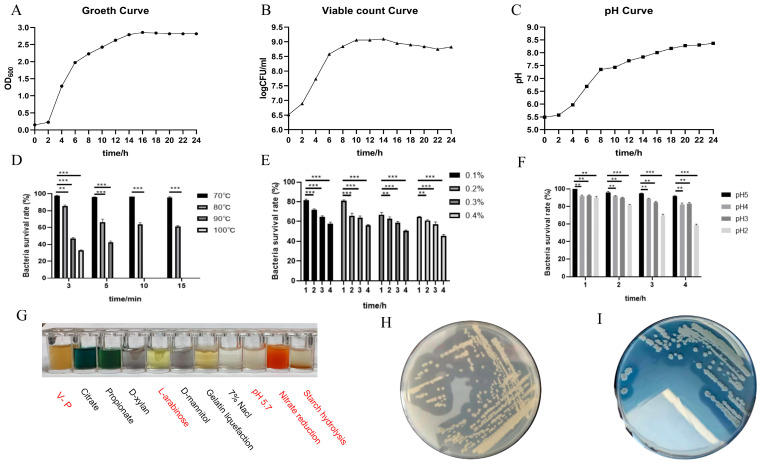
Probiotic properties of *B. velezensis* 411. (**A**–**C**) The growth curve, viable count curve, and pH curve of *B. velezensis* 411. (**D**) The temperature sensitivity of *B. velezensis* 411. (**E**) The bile salt tolerance of *B. velezensis* 411. (**F**) The acid tolerance of *B. velezensis* 411. (**G**) The biochemical reactions of *B. velezensis* 411. (**H**,**I**) The secretion ability of protease and amylase in *B. velezensis* 411. ** *p* < 0.01, *** *p* < 0.001.

The absorbance values of the cultures at OD_600_ exhibited a brief increase between 2 and 14 h, indicating that *B. velezensis* 411 was undergoing a logarithmic growth phase. Following 14 h, the absorbance curve gradually flattened and stabilized, suggesting that the cultures had entered the plateau phase (Figure 2A). No spore formation was observed under the specified culture conditions. This phenomenon may be attributed to the nutrient-rich medium and optimal growth parameters that favor nutrient proliferation over spore formation in *B. velezensis* 411. The total viable bacterial count rose sharply from 1 × 10^5^ CFU/mL to 1 × 10^9^ CFU/mL between 2 and 10 h, after which it tended to stabilize (Figure 2B). A notable increase in pH was observed during the first 10 h, followed by a decline in the rate of change. The strain demonstrated a clear ability to produce alkaline conditions, as evidenced by a pH value of 8.37 at the conclusion of the 24-h culture period (Figure 2C).

### 2.3. Resilience and Biochemical Properties of B. velezensis 411

The cell state of *B. velezensis* 411 was utilized for the tolerance test. *B. velezensis* 411 has strong temperature resistance: Its survival rate was more than 90 % after withstanding for 15 min at 70 °C, more than 50 % after withstanding for 15 min at 80 °C, higher than 40 % after 5 min at 90 °C, and at 32.26 % after 3 min at 100 °C (Figure 2D). The strain also has strong bile salt tolerance, with survival rates higher than 80 % after 1 h when bile salt was added at less than 0.2 %. Its survival rate was higher than 60 % at 2 h when bile salt was added at less than 0.4 %, and the survival rate of *B. velezensis* 411 was still close to 50 % at the limit of 4 h when bile salt was added at 0.4% (Figure 2E). *B. velezensis* 411 survived effectively in environments with a pH value above 3; however, at pH 2, the survival rate declined with prolonged incubation time, although it remained above 50% after 4 h (Figure 2F). In the basic biochemical tests, the V-P test, L-arabinose test, nitrate reduction, and starch hydrolysis showed positive results, and the rest were negative (Figure 2G). Enzyme production experiments using skim milk powder medium and starch medium verified that *B. velezensis* 411 possesses amylase and protease-producing properties (Figure 2H,I).

### 2.4. Drug Resistance and Antibacterial Activity B. velezensis 411

The results of the antibacterial activity assay demonstrated that *B. velezensis* 411 could effectively block the proliferation of *E. coli* K88, *E. coli* K99, *S. aureus* ATCC43300, *S. aureus* ATCC6538, *S. aureus* CVCC1882, *S. typhimurium* CVCC519, *S. typhimurium* ATCC14028, and *S. typhimurium* CMCC50115. The fermentation broth, suspension, and sediment were all inhibitory, with the fermentation broth having the strongest inhibitory effect (Table 1). This study tested 24 antibiotics from different broad categories (Table 2). Among these, *B. velezensis* 411 was found to be susceptible to 16 antibiotics, including tetracycline and erythromycin. Furthermore, it exhibited moderate sensitivity to furazolidone, cefoperazone, amikacin, and penicillin. However, *B. velezensis* 411 showed drug resistance to neomycin and three other antibiotics (Table 2).

### 2.5. Effects of B. velezensis 411 on Intestinal Tissues of Mice

**Figure 3:**
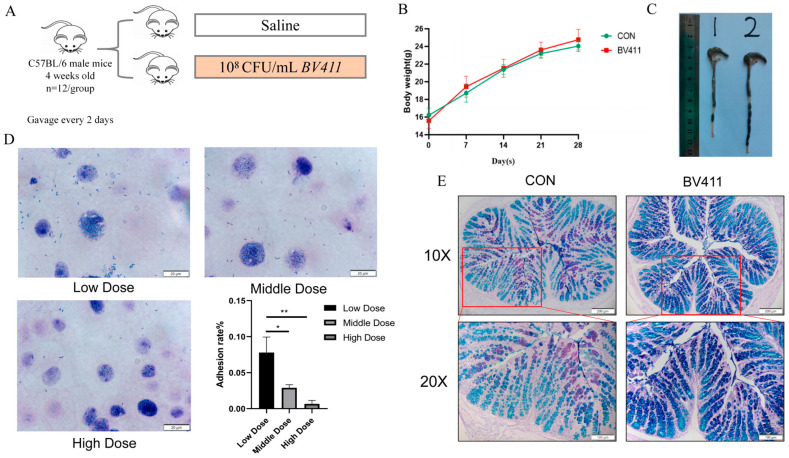
Effects of *B. velezensis* 411 on intestinal tissues of mice. (**A**) Experimental methods for *B. velezensis* 411 in mice. (**B**) The weight curve of mice. (**C**) The colon length of mice. (**D**) Adhesion of *B. velezensis* 411 to mice intestinal epithelial cells IEC-6. (**E**) (Alcian Blue-Periodic Acid-Schiff (AB-PAS) staining of mice colon. * *p* < 0.05, ** *p* < 0.01.

Four-week-old mice were divided into two groups of 12 mice each. One group was gavaged with saline, while the other group received a gavage of 10^8^ CFU/mL of cell state *B. velezensis* 411 bacterial solution at a dose of 150 µL every two days (Figure 3A). From day 7 until the end of the experiment, the body weights of mice in the *B. velezensis* 411 gavaged group were higher than those in the control group, but they were not statistically significant (*p* > 0.05, Figure 3B). On the 28th day of the experiment, dissection revealed that the colon length of mice in the *B. velezensis* 411 gavage group was generally higher than that of the control (*p* < 0.05, Figure 3C). Adhesion experiments of *B. velezensis* 411 were performed in vitro with mice intestinal epithelial cells IEC-6. Three gradients of low, medium, and high were set at 10^7^ CFU/mL, 10^8^ CFU/mL, and 10^9^ CFU/mL. The adhesion rate showed a significant decrease as the concentration of *B. velezensis* 411 increased (*p* < 0.05). At the concentration of *B. velezensis* 411 at 10^7^ CFU/mL, the adhesion rate was still less than 0.1% (Figure 3D). Furthermore, the colonic mucus secretion in mice treated with *B. velezensis* 411 was markedly higher than that in the control group, as demonstrated by (Alcian Blue-Periodic Acid-Schiff (AB-PAS) staining of colonic tissues (*p* < 0.05) (Figure 3E).

### 2.6. Effects of B. velezensis 411 on the Microbial Composition of the Mice Colon

**Figure 4:**
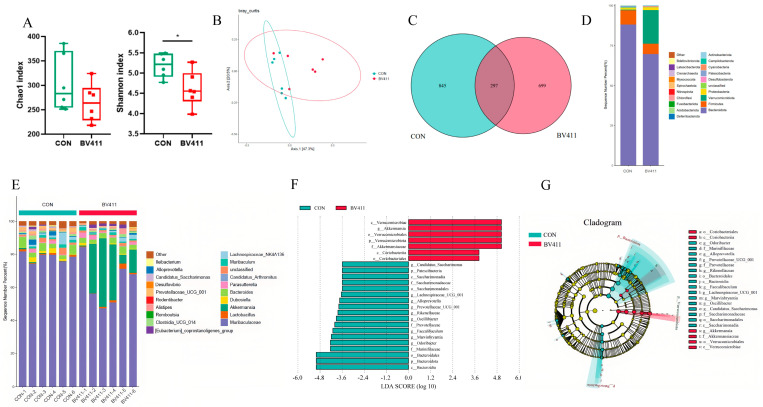
Effects of *B. velezensis* 411 on microbial c α-Diversity comparisons ((**A**) Shannon index and Chao1 index) between treatment and control groups; the data are displayed as mean ± SEM. (**B**) PCA (Principal component analysis) was used to assess the comparisons of β-diversity. (**C**) A Venn diagram was used to display the common species analysis. (**D**) The phylum-level community makeup of the gut microbiota. (**E**) Genus-level community makeup of the gut microbiota. (**F**) By applying an LDA (Linear Discriminant Analysis) score criterion of >2.0 and *p* < 0.05, LEFSe (Linear discriminant analysis Effect Size) was able to identify bacterial taxa in a differentiated manner. (**G**) Microbial composition cladogram of LEfSe (LDA score > 2, *p* < 0.05). * *p* < 0.05.

In the *B. velezensis* 411 group, both the Chao and Shannon indices exhibited a tendency to decline compared to the CON group, with the Shannon index drop being statistically significant (*p* < 0.05, Figure 4A). Principal component analysis (PCA) showed that while the colonic communities of the *B. velezensis* 411 group and the CON group differed in terms of diversity, they were not completely separable (Figure 4B). A comparison of common strains using a Venn diagram revealed 297 shared strains between the two groups (Figure 4C). Bacteroidetes was the most prevalent phylum in the colon, comprising two groups at the phylum level. Verrucomicrobia was ranked second in the *B. velezensis* 411 group, behind Bacteroidetes. Firmicutes was in the CON category (Figure 4D). Further analysis at the genus level showed that Muribaculaceae was the most represented genus in both the *B. velezensis* 411 group and the control group, accounting for 63.07% and 78.29% of the total. Figure 4E shows that the *B. velezensis* 411 group had a considerably higher representation of *Akkermansia* (20.97%) compared to the control group (0.36%, *p* < 0.05). Taxonomic analysis performed using LEfSe revealed that the genus *Akkermansia* within the family Verrucomicrobia had the most variation between the *B. velezensis* 411 group and the control group (*p* < 0.05, Figure 4F,G).

### 2.7. Effect of B. velezensis 411 in Feed on Growth Performance of Piglets

Probiotics are recognized for their significant roles in enhancing animal growth performance and improving feed conversion. In this study, supplementation with *B. velezensis* 411 notably improved the productive performance of weaned piglets. At the conclusion of the experiment, the body weight of the *B. velezensis* 411 group was significantly higher than the control at the end of the experiment (*p* < 0.05, Table 3). The average daily weight gain of the *B. velezensis* 411 group was higher than the control throughout the experimental phase (*p* < 0.05), with significant differences in the late and full phases of the experiment, and no significant differences in the early phase of the experiment (*p* > 0.05, Table 3). The average daily feed intake of the *B. velezensis* 411 group was slightly higher than the control during the experimental phase (*p* > 0.05, Table 3). In terms of the feed-to-weight ratio, the *B. velezensis* 411 group exhibited a lower ratio compared to the control group (*p* > 0.05, Table 3). Additionally, the diarrhea rate in the *B. velezensis* 411 group was significantly lower during the first 14 days of the experiment (*p* < 0.05, Table 3).

### 2.8. Effects of B. velezensis 411 on Intestinal Antioxidant Capacity of Piglet

The antioxidation indexes in colon mucosa of piglets were assessed (Table 4). The results indicated that the MDA content in the *B. velezensis* 411 group was significantly lower than that in the control group (*p* < 0.05). However, there was no significant difference in the T-AOC levels between the two groups (*p* > 0.05). As for the contents of SOD, the *B. velezensis* 411 group was significantly higher than the control (*p* < 0.05). There was no significant difference in the GSH content (*p* > 0.05, Table 4).

### 2.9. Effects of B. velezensis 411 on the Microbial Composition of the Piglet Colon

**Figure 5:**
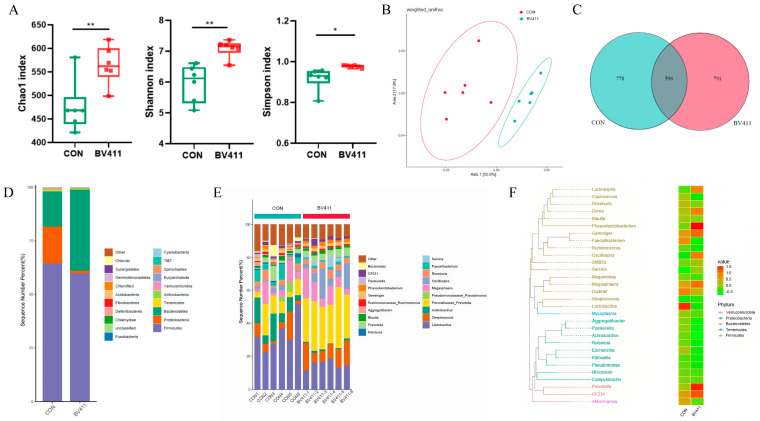
Effects of *B. velezensis* 411 microbial composition in piglet colon. (**A**) The data are presented as mean ± SEM, and the α-diversity comparisons were examined using Chao1, Shannon’s diversity, and the Simpson index. (**B**) PCA was used to assess the comparisons of β-diversity. (**C**) The Venn diagram displays the analysis of common species used. (**D**) The phylum-level community makeup of the gut microbiota. (**E**) Genus-level community makeup of the gut microbiota. (**F**) The phylogenetic tree and intergroup distribution heat map of the top 30 most abundant genera. * *p* < 0.05, ** *p* < 0.01.

The purpose of this analysis was to determine how *B. velezensis* 411 affected the composition of intestinal microbes in the colon chyme samples. Compared to the control group, the *B. velezensis* 411 group exhibited significant changes in the Chao, Shannon, and Simpson indices (*p* < 0.05, Figure 5A). This indicates that there were significantly differences in the colon community composition between the control and *B. velezensis* 411 treatments, as demonstrated by PCA diversity (*p* < 0.05, Figure 5B). A Venn diagram analysis revealed 596 common strains between the two groups (Figure 5C). In the *B. velezensis* 411 group, the relative abundance of Bacteroidetes was second only to that of Firmicutes. For Proteobacteria, the abundance of the control group was much higher than that of the *B. velezensis* 411 group (*p* < 0.05, Figure 5D). Further analysis at the genus level revealed that for *Lactobacillus*, *Streptococcus*, *Prevotellaceae_Prevotella*, and *Actinobacillus*, the total abundance of the four genera accounted for more than 50%, but the proportion of these four genera differed significantly in the two groups. The dominant genus in the control was *Lactobacillus* (27.97 %), but in the *B. velezensis* 411 group, the dominant genus was *Prevotellaceae_Prevotella* (21.65 %, Figure 5E). The phylogenetic tree and intergroup distribution heat map showed that the abundance of *Proteobacteria* was significantly lower in the *B. velezensis* 411 group compared to the control (*p* < 0.05, Figure 5F).

### 2.10. Effects of B. velezensis 411 on Transcriptome Analysis of the Piglet Colon

**Figure 6:**
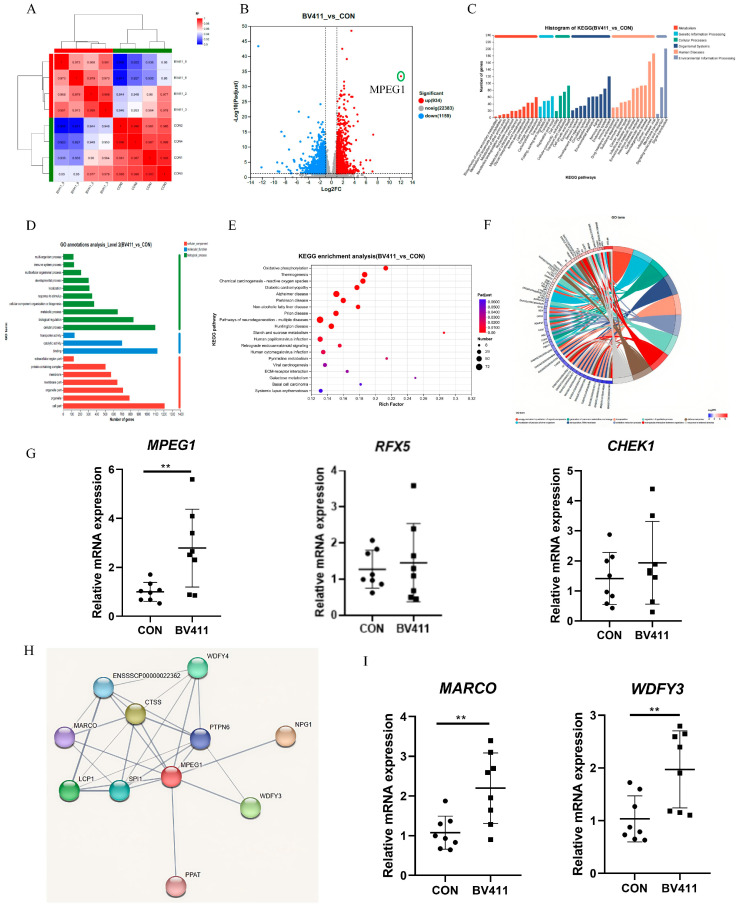
Effects of *B. velezensis* 411 on transcriptome analysis of piglet colon. (**A**) Relevance heat map. (**B**) Expression difference volcano map (Dashed lines: |LFC| > 1 (vertical), FDR-adjusted *p* < 0.05 (horizontal)). (**C**) KEGG classification map of genes that differ between samples. (**D**) GO classification statistics graph. (**E**) KEGG enrichment analysis bubble chart. (**F**) GO enrichment chord plot. (**G**) Differential expression of the genes. (**H**) Interacting protein prediction map for *MPEG1*. (**I**) Real-time quantitative PCR of upstream and downstream genes of *MPEG1*. ** *p* < 0.01.

The correlation among the four biological replicates for each sample is evident. The coefficients are distributed diagonally, indicating that the sequencing data exhibit strong reproducibility and high confidence, thereby supporting their use in subsequent differential expression analyses (Figure 6A). A total of 5477 differential genes were identified in the *B. velezensis* 411 group compared to the control group, of which 2668 were upregulated and 2809 were downregulated (Table A2). Additionally, the number of genes exhibiting a 2-fold difference in expression was assessed, with particular attention given to the significantly upregulated gene *MPEG1* (Figure 6B). *B. velezensis* 411 affects six KEGG metabolic pathways, including cell metabolism, genetic information processing, environmental information processing, cellular processes, organismal systems, and human diseases (Figure 6C). The GO classification statistics graph shows that more than 1000 upregulated genes or transcripts were assigned to cellular processes, binding, and cell parts (Figure 6D). The results of KEGG enrichment analysis showed a significant effect of *B. velezensis* 411 treatment group on starch and sucrose metabolism as well as pyrimidine metabolism compared to the control (Figure 6E). The GO enrichment chord plot showed that *MPEG1* was the most significantly changed gene (*p* < 0.05, Figure 6F). QPCR results confirmed a significant increase in the expression of *MPEG1* in the *B. velezensis* 411 group relative to the control (*p* < 0.05). However, the gene expressions of *RFX5* and *CHEK1* did not show significant differences when compared to the control (*p* > 0.05, Figure 6G). We created a protein interaction map of *MPEG1* on pigs showing 10 interacting proteins (Figure 6H). The gene expression of *MARCO* and *WDFY3* was significantly higher compared to the control (*p* < 0.05, Figure 6I).

## 3. Discussion

In large-scale livestock production, antibiotics have been routinely added to animal feed to enhance performance, particularly in pigs and chickens. However, the chronic misuse of antibiotics has resulted in the development of resistance, dysbiosis of intestinal flora in livestock and poultry, and even antibiotic-induced diarrhea [14,15]. Previous studies have identified probiotics as a promising alternative to antibiotics in animal feed. [16]. Common probiotics include *Lactobacillus*, *Saccharomyces*, *Bacillus*, etc. *Bacillus* has been developed as a representative of the probiotic class due to its advantages such as easy mass production, high survival rate, and relatively low market price [17]. Some *Bacillus* strains are also utilized as dietary supplements for humans due to their stable safety and even as agents for regulating intestinal health. As research progresses, new probiotics continue to be developed and explored. Notably, *B. velezensis* has garnered attention for its exceptional biocontrol capabilities. Recent studies have reported the use of *B. velezensis* as a feed additive to enhance livestock performance and intestinal health; however, the mechanisms through which *B. velezensis* affects monogastric animals, such as pigs and chickens, remain unclear [10].

Based on the aforementioned research background, a strain of *Bacillus* sp. was isolated and characterized from the intestine of a healthy pig. Reliable single colonies were obtained following several rounds of purification. A series of in vitro experiments were conducted to evaluate the potential of this *Bacillus* strain as a probiotic, including tests for temperature tolerance, bile salt and acid tolerance, antibiotic sensitivity, antimicrobial resistance, and growth characteristics. The experiments demonstrated the potential of this *Bacillus* strain as a probiotic. *Bacillus* spores exhibited the ability to endure low pH levels, while tolerance to transient high temperatures was attributed to the presence of feed plasmids. We performed 16S rRNA sequencing and whole-genome sequencing of the *Bacillus* strain. Based on the results of the 16S rRNA sequencing, we initially identified the isolated strain as *B. velezensis*. Furthermore, we analyzed the whole genome sequence by comparing it to the whole genome sequence of *B. velezensis* FBZ42 using ANI analyses, which yielded a similarity of 97.5%. Consequently, we confirmed that the strain was *B. velezensis* and designated it as *B. velezensis* 411. To enhance our understanding of *B. velezensis* 411, including its metabolites and metabolic pathways, we performed whole-genome sequencing. The sequencing results showed that it can secrete amylase and protease, which is consistent with the results of in vitro experiments. The secondary metabolites of *B. velezensis* 411 include several lipopeptides, such as locillomycin, bacillaene, fengycin, and difficidin, which elucidate the observed antibacterial properties in vitro. The results from pathogen–host interaction analysis indicated that *B. velezensis* 411 also inhibited the reproduction of pathogens such as *Streptococcus pneumoniae*, *Listeria monocy*, *Pseudomona*, and others that pose a risk to the healthy growth of livestock and poultry. The use of antibiotics can lead to the dissemination of resistance genes to other microbes in the gastrointestinal tract [18]. Among the 24 antibiotics tested, *B. velezensis* 411 showed resistance to only 4 antibiotics, a result that was also validated in the resistance gene prediction. Both in vitro experiments and the results of whole-genome sequencing confirmed the potential of *B. velezensis* 411 as a probiotic.

Based on the aforementioned results, we further conducted animal experiments using mice and piglets to evaluate the potential for in vivo applications. Previous reports indicate that antiadhesion tactics can successfully prevent diseases mediated by pathogens on the mucosal surface, particularly when combined with selection pressure for competitive rejection by healthy intestinal flora [19,20]. Cell adhesion assays using mouse intestinal epithelial cells IEC-6 showed that *B. velezensis* 411 exhibited minimal adherence to the epithelial cell surface. We observed a decreasing trend in the cell adhesion rate with elevated *B. velezensis* 411 addition, which may be related to the amount of additive. In the low-concentration range, the adhesion rate tends to increase with the rising number of bacteria. Conversely, in the middle- to high-concentration range, the adhesion rate may plateau or even decline. In very high concentration ranges, the adhesion rate significantly decreases due to metabolic toxicity and limitations imposed by physical spatial constraints [21]. In the present study, the adhesion rate of *B. velezensis* 411 was found to be less than 0.1%, indicating minimal adherence to the cell surface. It has also been reported that high concentrations of *bacilli* induce quorum sensing, which leads to a decrease in the adhesion rate [22]. Consequently, we hypothesize that the strategy employed by *B. velezensis* 411 to improve microflora homeostasis in the animal gut may not be related to the adhesion effect. After gavage experiments on mice, AB-PAS staining of colonic tissues demonstrated that colonic mucin secretion was significantly higher in mice gavaged with *B. velezensis* 411 compared to control group. Combined with the results of microbiome sequencing of colonic chyme, we hypothesized that this increase is associated with a significant rise in *Akkermansia*. This is why *Akkermansia* is considered a model of next-generation probiotics [23]. It has been reported that the identification of a lipid from the cell membrane of *A. muciniphila*’s recapitulates the immunomodulatory activity of *A. muciniphila* in cell-based assays [24]. Although the specific mechanism underlying *B. velezensis* 411-mediatedincrease in *Akkermansia* abundance remains unverified, data indicate that *B. velezensis* 411 shifts the gut microbiota toward a healthier composition. The increase in intestinal mucus layer thickness in mice was accompanied by an increase in the proportion of *Akkermansia muciniphila* [25]. This is also consistent with the phenomenon we observed.

The enhancing effect of *B. velezensis* 411 on animal growth performance was more pronounced in our experiments with piglets as the animal model compared to mice, which may be attributed to species differences. In addition to significantly increasing the body weight and average daily gain of piglets, *B. velezensis* 411 also effectively reduced the rate of diarrhea in piglets. Diarrhea, particularly due to early weaning, poses a significant challenge in intensive farming [26]. Contributing factors include incomplete intestinal development, unstable intestinal microbial composition, and antinutritional factors present in feed at the young age of piglets [27,28]. Numerous studies have shown that improved animal production performance is associated with improved intestinal absorption, such as increased intestinal villi height and crypt depth [29]. However, our study did not observe significant alterations in intestinal villi and crypt depth. The effectiveness of *B. velezensis* 411 as a probiotic against diarrhea significantly increases the significance of further research on its mechanism of action.

Previous studies have indicated a significant correlation between diarrhea in piglets and oxidative stress. Consequently, we assessed the relevant indicators of oxidative stress, and our results showed that the antioxidant capacity of piglets improved. It is reported that certain probiotic strains, such as *Bifidobacterium*, *Lactobacillus*, and *Bacillus*, exhibit strong antioxidant capacity, which can mitigate oxidative stress damage both in vivo and in vitro [30]. Certain probiotics can enhance tissue antioxidant capacity by scavenging reactive oxygen species [31,32]. Among the most significant antioxidant enzymes in lactic acid bacteria are superoxide dismutases (SODs), which dismutate O^2−^, thereby reducing the intracellular concentration of free metal cations and mitigating the damage caused by H_2_O_2_. Manganese (Mn), iron (Fe), and copper (Cu) are the primary metal cofactors necessary for the enzymatic function of SODs [31]. Additionally, it has been reported that *Pediococcus pentosaceus* alleviates MPTP-induced oxidative stress by regulating the gut microbiota–gut–brain axis [33]. Furthermore, numerous studies indicate that *Bacillus subtilis* mitigates intestinal oxidative injury through the Nrf2-Keap1 pathway [34]. While studies have identified some *Bacillus* strains are suitable for preventing oxidative stress, the underlying mechanisms remain largely unknown [17]. In the PCoA analysis of the gut microbiome in piglets, the intestinal flora of the *B. velezensis* 411 treated group was found to be significantly different from that of the control group. Further analysis revealed that the abundance of the Proteobacteria phylum was significantly lower in the *B. velezensis* 411 treated group than in the control group. Some studies have explored the association between an abnormal expansion of Proteobacteria and a compromised ability to maintain a balanced gut microbial community [35]. These studies suggest that an increased prevalence of Proteobacteria is a potential diagnostic signature of dysbiosis and risk of disease [36].

In a study of the piglet intestinal transcriptome, we observed widespread upregulation and downregulation of numerous genes. However, based on the significance of pathway enrichment and their fundamental biological relevance, we prioritized the *MPEG1*, *RFX5*, and *CHEK1* genes for focused analysis. In parallel, qPCR experiments were performed to verify the results of these gene upregulations. Experiments showed that the *MPEG1* genes were significantly upregulated, while the remaining two genes were not significantly changed. The *MPEG1* gene is predominantly expressed in macrophages, and the protein it encodes may play a crucial role in the activation, differentiation, and functional regulation of these immune cells. Macrophages are essential components of the immune system, tasked with the clearance of pathogens, apoptotic cells, and the facilitation of tissue repair [37]. Consequently, we hypothesize that the upregulation of *MPEG1* gene expression signifies an enhancement in the body’s capacity to eliminate pathogens [38]. This result mirrors the reduction in the abundance of pathogenic bacteria observed in the intestine of piglets. Consequently, we hypothesize that the increase in piglet production performance may be associated with an increase in immune function. Additionally, there are reports suggesting that *MPEG1* is not crucial for antibacterial or antiviral immunity, but it plays a role in antigen presentation [39]. There is substantial evidence that the upregulation of *MPEG1*, within a specific range, positively influences the organism. Furthermore, an analysis of the interacting proteins of *MPEG1* was conducted. Recent studies have shown that supplementation with probiotics and their associated metabolites correlates with the upregulation of *MPEG1*. However, the operative mechanisms underlying this relationship require further characterization [40,41]. *MARCO* is a co-expressed gene of *MPEG1* [42], while *WDFY3* functions as a downstream regulator of *MPEG1* [43]. We included both genes for parallel qPCR detection. *MARCO* is a macrophage receptor characterized by a collagenous structure. The protein encoded by the *MPEG1* gene is a member of the class a scavenger receptor family and is part of the innate antimicrobial immune system, which can bind both Gram-negative and Gram-positive bacteria via an extracellular, C-terminal, scavenger receptor cysteine-rich (SRCR) domain. The MPEG1 protein may form a trimeric molecule via the association of the collagenous domains of three identical polypeptide chains [42]. *WDFY3*, as the downstream gene of *MPEG1*, encodes a protein that binds to phosphatidylinositol 3 phosphate, acting as a master conductor for autophagic clearing of aggregates [44]. Collectively, these data suggest that *B. velezensis* 411 may exert its antibacterial function through crosstalk with the intestinal mucosa and the *MPEG1* signaling pathway.

## 4. Materials and Methods

### 4.1. Animal Ethics

All procedures in this study received approval from the China Agricultural University Laboratory Animal Welfare and Animal Experimental Ethical Inspection Committee (AW20603202-1-2; AW20603202-1-3).

### 4.2. Bacterial Isolation and Identification

In this study, fresh pig feces were diluted with physiological saline and spread onto LB plates. After 24 h of incubation at 37 °C, single colonies were obtained and purified three times. Genomic DNA was extracted from Bacillus using Bacteria Genomic DNA Kit (CW Biotech, Beijing, China). Primer sets 27F (5′-AGAGTTTGATCMTGGCTCAG-3′) and 1492R (5′-GGTTACCTTGTTACGACTT-3′) were used to amplify the DNA fragments, which were then subjected to sequencing (Qingke, Beijing, China). The result was further compared with the NCBI sequence database by the basic local alignment search tool (BLAST). Whole-genome sequencing of bacterial completion maps was achieved by second-generation sequencing overlaid with Illumina Hiseq + PacBio sequencing. Each sample provides not less than 100 × genome and 100 × Illumina sequencing data to ensure a complete and accurate assembly. The completion map can avoid the loss of information of small plasmids (<15 kb) and ensure the acquisition of a complete genome containing plasmids. The study is registered under the accession number PRJNA977461.

### 4.3. Animal Experiment

In total, 24 male C57BL/6 mice that were twenty-eight days old and of similar weight were split at random into control (CON, 150 μL normal saline) and the *B. velezensis* 411 supplementation group (BV411, 150 μL 1 × 10^8^ CFU/mL) [45,46]. Normal saline or BV411 was administrated by gavage every other day for 28 days. All mice had similar initial body weights (16–18 g) and were fed with the same diet with free access to water ad libitum. The animals were housed at 24 °C with the humidity maintained at 55%, and a daily light cycle of 12 h of light followed by 12 h of darkness was implemented.

Ninety-six healthy 28-day-old crossbred piglets (Duroc × Landrace × Yorkshire), sourced from commercial piglet production farms, with an initial body weight of 9.2 ± 1.68 kg were used. Piglets were randomly assigned to 2 treatment groups according to their body weights. They were housed in an environmentally controlled facility maintained at 22 °C and 45% relative humidity, with a stocking density of 0.6 m^2^ per piglet. Each treatment group consisted of six replicates (pens), with eight piglets per replicate. The experimental diets were as follows: (1) CON group: basic diet (see Table A3); (2) BV411 group: basic diet supplemented with *B. velezensis* 411 at 5 × 10^7^ CFU/kg feed [47]. The nutritional composition of the basic diet met or exceeded the requirements established by the National Research Council (NRC, 2012).

### 4.4. Sample Collection

At the conclusion of the animal studies, blood samples were collected from the eye sockets of 12 mice, after which the mice were euthanized. Approximately 1 cm segments of the jejunum, ileum, and colon were excised and fixed in 4% paraformaldehyde (PFA) overnight for subsequent morphological analysis. The remaining intestinal tissues and chyme samples were snap-frozen using liquid nitrogen and stored at −80 °C for future studies. Piglet samples were collected in a manner analogous to that used for the mice.

### 4.5. Analysis of Nutrient Composition in Feed

The samples were grounded to pass through a 1 mm (40 mesh) screen. Feed samples were analyzed for crude protein (CP) (method 984.13), dry matter (DM) (method 930.15), and ash (method 942.05) using the AOAC [48]. Organic matter (OM) was calculated as the difference between dry matter (DM) and ash. Gross energy (GE) was determined with an Automatic Isoperibol Oxygen Bomb Calorimeter (Parr 6400 Calorimeter, Moline, IL, USA). Neutral detergent fiber (NDF) and acid detergent fiber (ADF) were determined using the fiber bags and fiber analyzer equipment (Ankom Technology, Macedon, NY, USA). The calcium content was determined based on GB/T6436-2018 [49]. The total phosphorus content was determined according to the GB/T6437-2018. Feed samples were dissolved using 1 mL of 0.02 mol/L HCl solution and filtered through a 0.45 μm membrane prior to analysis of hydrolyzed amino acid (AA) content using an automatic amino acid analyzer [50] (L-8900; Hitachi, Tokyo, Japan).

### 4.6. Growth Characteristics

A single colony of *B. velezensis* 411 was selected and inoculated into LB liquid medium, followed by cultivation at 37 °C. Subsequently, the LB liquid medium was infected with activated *B. velezensis* 411 at a ratio of 1% while maintaining the same culture conditions. Every two hours, samples were taken to count the viable bacteria for a full day and analyze the pH and OD_600_ values.

### 4.7. Acid and Bile Salt Resistant

Tolerance to acid and bile salts was evaluated by culturing the 1% activated BV411 in sterile LB liquid medium with different pH (2.0, 3.0, 4.0, 5.0) and with different bile salts (0.1%, 0.2%, 0.3%, 0.4%). The viable bacteria number were recorded every hour for 4 h with the spread plate method. Each experiment was performed in triplicate.

### 4.8. High-Temperature Resistance

Aliquots of *B. velezensis* 411 incubated for 12 h were placed at 70 °C, 80 °C, 90 °C, and 100 °C for 3, 5, 10, and 15 min. The viable counts were recorded, with the counts at 0 min serving as controls for calculating survival rates. Each experiment was repeated three times.

### 4.9. Antibiotic Sensitivity Assay

Twenty-four representative antibiotics, including amikacin, gentamicin, cefradine, and tetracycline, were selected from each class to test the drug sensitivity of *B. velezensis* 411 using drug-sensitive paper tablets. The tablets were spread on a flat surface and compacted gently. The plates were then incubated at 37 °C for 24 h. The criterion for antibiotic sensitivity was based on the diameter of the antibiotic circle, where <15 mm was considered as resistant, 16–20 mm as moderate sensitive, and greater than 20 mm as sensitive.

### 4.10. Biochemical Analysis

The detection of protease was achieved using a nonfat dry milk medium, while amylase detection was performed using a starch-selective medium. The *Bacillus* biochemical test reagent strips (Qingdao Hope Bio-Technology Co., Qingdao, China) were utilized for various tests, including the Voges–Proskauer (V-P) test, citrate test, and propionate test.

### 4.11. Bacteriostasis Test

A single colony of activated indicator bacteria was selected and inoculated into LB medium. The mixture was thoroughly mixed and cultured at 37 °C with 220 rpm agitation for 12 h to reach the concentration at 1 × 10^8^ CFU/mL. Proportionately, 1% B. velezensis 411 was inoculated for 18 h following by centrifugation at 3500× *g* for 15 min at 4 °C. The supernatant was collected, and the bacteria were resuspended in an appropriate amount of sterile saline. Finally, 150 μL of samples was then applied to the Oxford cup and cultured at 37 °C for 12 h.

### 4.12. Adherence to IEC-6 Cells

The *B. velezensis* 411 pellets obtained from bacteriostasis test were washed three times with PBS and resuspended in DMEF/12 without serum or antibiotics with concentrations at 1 × 10^7^, 1 × 10^8^, and 1 × 10^9^ CFU/mL. Following the incubation period, the supernatant was disposed, and the cells were subjected to three washes with PBS to ensure the removal of any residual bacteria. Then, 1 mL 0.1% Triton-100 was added to lyse the cells for 15 min to release the bacteria. Lysate was then transferred to a centrifuge tube. Moreover, washed cells after 2 h of co-culturing were fixed with formaldehyde for 30 min for further Gram staining to visualize the adhesion of the *B. velezensis* 411 to IEC-6 cells under co-culturing.

### 4.13. Histological Analysis

Intestinal tissues fixed in 4% paraformaldehyde (PFA) were embedded in paraffin and sectioned to a thickness of 3 μm. Subsequently, these sections underwent hematoxylin and eosin staining. To visualize goblet cells, the sectioned intestinal tissues were subjected to Alcian blue and periodic acid–Schiff (PAS) staining after being dehydrated using a gradient of alcohol and xylene. Images were captured using the same microscope.

### 4.14. Microbiota Profiling

Genomic DNA from colon chyme was extracted using commercial kits. The extracted genomic DNA was subsequently amplified and subjected to paired-end sequencing on the Illumina MiSeq platform. The DADA2 method recommended by QIIME2 (2023.12) was used for quality control of raw data. The quality-controlled data were then subjected to OTU clustering/de-noising and species classification analysis to generate the species abundance spectrum and other species classification grades. Based on this, the abundance and diversity index of OTU were further analyzed. At each taxonomic level, by mapping to Silver database, the community structure of species annotation was generated. Principal coordinate analysis (PCoA) was also performed to obtain principal coordinates among microbiota and visualize the intricate data. The raw datasets are available at GenBank: PRJNA977815.

### 4.15. Antioxidant Indexes of Piglet Colon

A total of 0.1 g colon mucosal was placed in 1.5 mL Eppendorf tube with 900 μL sterile normal saline and three small steel balls. The tissue was homogenized using a tissue crusher to produce a 10% tissue homogenate. The homogenate was then centrifuged at 3500× *g*, 4 °C, for 15 min. Carefully transfer the supernatant to a new Eppendorf tube to measure the volume and the protein concentration by the BCA method. A commercial kit was employed to evaluate the antioxidant capacity of the collected supernatant.

### 4.16. Transcriptomic Analysis

Colonic mucosas from both control and treated piglets were used for transcriptome analysis. RNA with a high integrity number was used for cDNA library construction using the TruSeq™ Stranded Total RNA Library Prep Kit reagents (Shenggong Biotech, Shanghai, China). All procedures were conducted in accordance with the manufacturer’s instructions. The constructed cDNA library was the sequenced by Illumina HiSeq platform. After the quality control of raw data, differentially expressed genes (DEGs) screening and bioinformatics analysis were performed using DESeq2 (Version 1.24.0) software. The DEGs were identified with a threshold of |log2 (fold change)| ≥ 1 and *p*-value < 0.05. The Gene Ontology (GO) database and the Kyoto Encyclopedia of Genes and Genomes (KEGG) database were employed for further biological function annotation. The raw data of RNA sequencing have been deposited in the NCBI Sequence Read Archive (SRA) database (PRJNA1179404).

### 4.17. RNA Extraction and Quantitative Real-Time PCR

Total RNA was extracted from frozen intestinal tissues using TRIzol reagent (Invitrogen, Carlsbad, CA, USA) according to the protocol as previously described [51]. cDNA was generated by reverse transcription of RNA with commercial reverse transcription kit (Mei5bio, Beijing, China). qPCR reactions were performed with SYBR Green PCR Supermix (Mei5bio, Beijing, China) and specific primers (Shenggong Biotech, Shanghai, China), as well as a run on a Real-Time PCR System (Roche LightCycler96, Munich, Germany) (Table A4).

### 4.18. Data Analysis

SAS version 9.1 was utilized to conduct the statistical analysis. Statistical significance was assessed using ANOVA and Tukey’s multiple-range tests. The results are presented as Mean ± SEM. *p* < 0.05 was considered a significant difference; *p* < 0.01 and *p* < 0.001 were considered highly significant differences. GraphPad Prism 9.5 and Origin 2021 software were employed for graph plotting.

## 5. Conclusions

In summary, our data suggest that supplementation with *B. velezensis* 411 can improve intestinal health and antioxidant capacity in animal models, as well as enhance the gut microbial composition of animals. The potential mechanisms by which dietary *B. velezensis* exerts its effects are attributed to the upregulation expression of the MPEG1 pathway. This study confirms the probiotic effect of *B. velezensis* 411 in piglets and provides a theoretical basis for the application of *B. velezensis* as a feed additive in weaned piglets.

## Figures and Tables

**Table 1:** Antibacterial activity of *B. velezensis* 411.

Indicator Species	Inhibitory Zone (mm)		
	Fermentation Broth	Sediment	Bacterial Suspensions
*S. aureus* ATCC6538	17.5	16.5	15.0
*S. aureus* CVCC43300	16.0	14.0	14.0
*S. aureus* CVCC1882	19.0	16.5	15.0
*E. coli* K99	14.0	15.5	13.5
*E. coli* K88	15.0	15.0	13.0
*S. typhimurium* CVCC519	17.0	15.0	14.0
*S. typhimurium* ATCC 14028	16.0	14.0	12.0
*S. typhimurium* CMCC 50115	16.0	14.5	12.0

**Table 2:** Antibiotic sensitivity of *B. velezensis* 411.

Antibiotic Names	Content (μg/pill)	Inhibition Zone (mm)	Sensitivity
Cefradine (RAD)	30	45	sensitive
Carbenicillin (CB)	100	35	sensitive
Norfloxacin (NOR)	10	33	sensitive
Tetracycline (TE)	30	23	sensitive
Erythromycin (E)	15	23	sensitive
Minocycline (MI)	30	40	sensitive
Ceftriaxone (CTR)	30	37	sensitive
Kanamycin (K)	30	33	sensitive
Midecamycin (MD)	30	25.5	sensitive
Cefazolin (CZ)	30	52	sensitive
Cefuroxime (CXM)	30	41	sensitive
Ciprofloxacin (CIP)	5	43	sensitive
Doxycycline (DX)	30	33	sensitive
Ampicillin (AM)	10	26	sensitive
Piperacillin (PIP)	100	28	sensitive
Ceftazidime (CAZ)	30	30	sensitive
Penicillin (P)	10 U	17	moderate sensitive
Amikacin (AK)	30	20	moderate sensitive
Cefoperazone (CFP)	75	17	moderate sensitive
Furazolidone (FZ)	300	20	moderate sensitive
Neomycin (N)	30	10	drug resistance
Sulfamethoxazole (SXT)	23.75	0	drug resistance
Ofloxacin (OFX)	5	0	drug resistance
Gentamicin (GM)	10	0	drug resistance

Note: A bacteriosphere diameter of more than 20 mm is sensitive, while a circle diameter of less than 15 mm is resistant, and 16–20 mm indicates moderately sensitive.

**Table 3:** Effect of *B. velezensis* 411 on growth performance of piglets.

Items	Treatments		SEM	*p*-Value
	CON	BV411		
BW, kg				
0 d of age	9.28	9.34	0.039	0.456
7 d of age	11.87 ^b^	12.17 ^a^	0.070	0.018
14 d of age	16.19	16.54	0.116	0.135
28 d of age	19.76 ^b^	21.35 ^a^	0.327	0.007
0–14 d of age				
ADG, kg	0.28	0.31	0.009	0.102
ADFI, kg	0.63	0.66	0.011	0.127
FCR	2.24	2.16	0.057	0.498
Diarrhea rate, %	15.48	9.97	1.253	0.019
15–28 d of age				
ADG, kg	0.33 ^b^	0.38 ^a^	0.320	<0.001
ADFI, kg	0.76	0.79	0.020	0.540
FCR	2.28	2.06	0.067	0.097
0–28 d of age				
ADG, kg	0.32 ^b^	0.36 ^a^	0.008	0.004
ADFI, kg	0.69	0.73	0.013	0.257
FCR	2.17	2.02	0.051	0.146

Note: BW = body weight; ADG = average daily gain; ADFI = average daily feed intake; FCR = feed conversion ratio (feed intake/body weight gain, kg/kg). ^a,b^ Values within a row with no common superscripts differ significantly (*p* < 0.05).

**Table 4:** Antioxidant effect of *B. velezensis* 411 on colon in piglets.

Items	CON	BV411	SEM	*p* Value
T-SOD (U/mgprot)	10.12 ^b^	14.11 ^a^	0.331	0.014
T-AOC (U/mgprot)	1.86	2.32	0.314	0.482
MDA (nmol/mgprot)	2.37 ^a^	1.53 ^b^	0.162	0.032
GSH (mg/g)	5.52	5.76	0.827	0.851

Note: SEM means standard error of the mean; different letter superscripts in the same row indicate a significant difference (*p* < 0.05); n = 6.

## Data Availability

The data that support the findings of this study are available from the corresponding author upon reasonable request. The datasets supporting the conclusions of this article are available in the NCBI Sequence Read Archive (SRA) repository under accession number PRJNA977461, PRJNA977815 and PRJNA1179404.

## Appendix A

**Table A1:** Gene prediction statistics.

Gene Number	Total Length (bp)	Average Length (bp)	GC% (Gene Region)	Gene Length/Genome (%)	Gene Density
3662	3,445,578	940.90	47.42	88.61	0.94

**Table A2:** Differential gene expression statistics table.

Group	Total DEG	Up	Down
BV411_vs_CON	5477	2668	2809

**Table A3:** Composition and nutrient content of basal diets (air-dry basis).

Items	Contents, %
Ingredient	
Corn	58.60
Soybean meal	13.00
Whey powder	8.00
Fish meal	3.00
Soy protein concentrate	5.00
Extruded full-fat soybean	5.00
Glucose	2.00
Limestone	0.80
Dicalcium phosphate	1.00
NaCl	0.20
DL-methionine	0.10
L-Lysine-HCL	0.55
L-Threonine	0.20
L-Tryptophan	0.05
Soybean Oil	2.00
Premix ^1^	0.50
Nutrient level ^2^	
DE, MJ/kg	14.82
NE, MJ/kg	11.38
OM	88.96
NDF	9.56
ADF	3.38
CP	18.86 (18.95)
Calcium	0.8 (0.84)
Total phosphorus	0.65 (0.67)
Threonine	0.95
Lysine	1.53
Methionine	0.44
Tryptophan	0.26

^1^ Premix provided the following per kg of complete diet for growing pigs: vitamin A, 12,000 IU; vitamin D3, 2500 IU; vitamin E, 30 IU; vitamin K_3_, 3.0 mg; vitamin B_12_, 0.012 mg; riboflavin, 4.0 mg; pantothenic acid, 15.0 mg; niacin, 40.0 mg; choline chloride, 400.0 mg; folic acid, 0.7 mg; vitamin B_1_, 1.5 mg; vitamin B_6_, 3 mg; biotin, 0.1 mg; Mn, 40.0 mg; Fe, 90.0 mg; Zn, 100.0 mg; Cu, 8.8 mg; I, 0.35 mg; Se, 0.3 mg. ^2^ The values in parentheses are the analyzed values of nutrient levels in the experimental basal diets. Others are calculated values. DE = digestible energy; OM = organic matter; CP = crude protein; NDF = neutral detergent fiber; ADF = acid detergent fiber; NE = net energy.

**Table A4:** Primers used in this study.

Primers	Sequences (5′ to 3′)	Product Length (bp)	Accession No.
WDFY3	F: CTACCTCTAGCAGAACGCCG	205	XM_021101795.1
	R: GGCGAGAGATGGTCATGAGG		
MARCO	F: AAGGCCCACCAGGAATCAAG	109	NM_001256366.1
	R: AAGTCACCTTTATGCCCCCG		
CHEK1	F: ACTTCCGGCTTTCTAAGGGTG	390	XM_005667481.3
	R: CCACCACGTGGGTCTCAAAT		
MPEG1	F: GCTGTCAAAGACGGTGGAGA	321	NM_001267892.1
	R: GAACACTCCAGGCGGTTGTA		
RFX5	F: GATGAGCCTGATGCTAAGAGC	134	XM_003125766.4
	R: CCCTCTACTTTGTTCTGCACG		
